# The Specificity of Observational Studies in Physical Activity and Sports Sciences: Moving Forward in Mixed Methods Research and Proposals for Achieving Quantitative and Qualitative Symmetry

**DOI:** 10.3389/fpsyg.2017.02196

**Published:** 2017-12-19

**Authors:** M. Teresa Anguera, Oleguer Camerino, Marta Castañer, Pedro Sánchez-Algarra, Anthony J. Onwuegbuzie

**Affiliations:** ^1^Faculty of Psychology, Institute of Neurosciences, University of Barcelona, Barcelona, Spain; ^2^INEFC (National Institute of Physical Education of Catalonia), IRBLLEIDA (Lleida Institute for Biomedical Research Dr. Pifarré Foundation), University of Lleida, Lleida, Spain; ^3^Department of Statistics, Faculty of Biology, University of Barcelona, Barcelona, Spain; ^4^Department of Educational Leadership and Counseling, Sam Houston State University, Huntsville, TX, United States; ^5^Faculty of Education, University of Johannesburg, Johannesburg, South Africa

**Keywords:** systematic observation, qualitative recording transformation, qualitative-quantitative integration, qualitative-quantitative symmetry, sport and physical activity sciences

## Abstract

Mixed methods studies are been increasingly applied to a diversity of fields. In this paper, we discuss the growing use—and enormous potential—of mixed methods research in the field of sport and physical activity. A second aim is to contribute to strengthening the characteristics of mixed methods research by showing how systematic observation offers rigor within a flexible framework that can be applied to a wide range of situations. Observational methodology is characterized by high scientific rigor and flexibility throughout its different stages and allows the objective study of spontaneous behavior in natural settings, with no external influence. Mixed methods researchers need to take bold yet thoughtful decisions regarding both substantive and procedural issues. We present three fundamental and complementary ideas to guide researchers in this respect: we show why studies of sport and physical activity that use a mixed methods research approach should be included in the field of mixed methods research, we highlight the numerous possibilities offered by observational methodology in this field through the transformation of descriptive data into quantifiable code matrices, and we discuss possible solutions for achieving true integration of qualitative and quantitative findings.

Diverse substantive areas have increasingly found their way into the expanding epistemological and methodological arsenal applied in mixed methods research in recent years (Ivankova and Kawamura, 2010). Mixed methods studies have been defined by several authors as studies aiming to integrate qualitative and quantitative elements. Johnson et al. (2007, p. 123), after analyzing 19 definitions provided by experts in the field, proposed the following definition: “Mixed methods research is the type of research in which a researcher or team of researchers combines elements of qualitative and quantitative research approaches (e.g., use of qualitative and quantitative viewpoints, data collection, analysis, inference techniques) for the broad purposes of breadth and depth of understanding and corroboration.” Empirical studies undertaken in the field of sport and physical activity have traditionally largely overlooked the methodological—and epistemological—opportunities offered by mixed methods research designs, but a growing number of studies in the field of sport and physical activity have shown the enormous potential that these designs offer for studying behaviors related to individual performance (Camerino et al., 2012c; Iglesias and Anguera, 2012), team performance (Camerino et al., 2012b,c,d,e), use of laterality and motor skills (Castañer et al., 2012), and use of sports facilities by children (Pérez-López et al., 2016), to name but a few examples. Settings of this type contain an enormous conceptual richness to be explored and methodologically captured, and we believe that the time has come to build on lessons learned and continue to move forward.

Although observation and other sources of data have been given some attention in the mixed methods research literature, few researchers have applied true observational research methods. Systematic observation is a scientific procedure for analyzing perceivable behaviors that occur spontaneously in a natural setting (Bakeman and Gottman, 1997; Anguera, 2003). In recent years, however, there has been a surge in the number of empirical studies involving the application of mixed methods research designs rooted in systematic observation in the field of sport and physical activity (Camerino et al., 2012a; Anguera et al., 2014). For this reason we believe that it is time to reconsider studies that apply systematic observation through a mixed method design in sport and physical activity. Examples include shots in soccer (Maneiro et al., 2017), handball (Freitas et al., 2010), or basketball (Fernández et al., 2009), corner kicks and throw-ins (Casal et al., 2015), symmetry of actions and reactions in fencing (Tarragó et al., 2017), maneuvers in synchronized swimming (Rodríguez-Zamora et al., 2014), errors in judo (Gutierrez-Santiago et al., 2013), pace during track events (Aragón et al., 2017), influence of ball size on children's performance in basketball (Lapresa et al., 2013a), use of gestures and signals by coaches and physical education teachers (Castañer et al., 2013), and compliance with rules and regulations, which themselves serve as a reference framework. The key to accurately capturing these realities lies in the application of an observational methodology that consists of the following successive stages: construction of an *ad-hoc* observation instrument, computerized recording and coding of behaviors observed, data quality control, and quantitative analysis of resulting datasets using adequate techniques for obtaining structured categorical data (in particular, lag sequential analysis, polar coordinate analysis, and T-pattern detection). Each of these techniques is governed by methodological rigor and scientific logic (Portell et al., 2015a).

Many studies portrayed as representing mixed methods research studies are constrained by diverse methodological shortcomings. However, in our opinion, there are two major ones: inadequate integration of qualitative and quantitative data and a lack of symmetry between the two approaches. Greater symmetry between quantitative and qualitative approaches is methodologically desirable given the need to merge both perspectives, although there are obviously situations in which a greater emphasis on one approach or another is preferable (Sandelowski et al., 2009). There are two distinct approaches to asymmetry within the theoretical framework. The first is a phenomenological approach, or more specifically, an “enactive or radical-embodiment” approach to the neuroscience of consciousness (Thompson and Varela, 2001; Lutz et al., 2002). This approach involves integrating first-person (phenomenological) data with neuroimaging data in order to explore the mutual constraints between these two types of data described in a different manner. The phenomenological approach is used in cluster trials where physiological data are obtained from participants in experimental situations. The second approach, traditionally viewed as more complex, is the successful mixing of qualitative and quantitative elements. We believe that the complexity of this approach lies in the nature of the data involved and it requires robust solutions to strike a balance between the qualitative and quantitative elements.

Researchers of systematic observation in the field of sport and physical exercise fundamentally draw their data from what could be considered exemplary sources, namely video or sound recordings of behaviors (i.e., direct observation; Anguera, 2003) and narratives from in-depth interviews (i.e., indirect observation; Morales-Sánchez et al., 2014; Anguera et al., 2017). Less frequently, they use elicited responses (i.e., responses to structured or semi-structured interviews –Arias and Anguera, 2017- or questionnaires), simulated data (Manolov and Losada, 2017), and physiological data (Zurutuza et al., 2017). Our aim in this article, then, is to provide guidance on how to resolve two of the main shortcomings that undermine mixed methods research in the field of sport and physical activity—integration and symmetry of qualitative and quantitative data—and to show how these solutions could be extrapolated to other fields. In the following sections, we discuss three fundamental concepts with the aim of contributing to the ongoing dialog in mixed methods research and helping this field to advance.

## Sport and physical activity as a new substantive area in mixed methods research

In the late 1990s, Biddle (1997) found very little diversity in research methods used in empirical studies in two of the most prestigious sport and physical activity journals he chose to study—*The Journal of Sport and Exercise Psychology* (JSEP), a leading research journal in the field, and *The International Journal of Sport Psychology* (IJSP), which was the first journal in this field. Most of the quantitative research was based on regression techniques and discriminant analysis, while most of the qualitative research drew on interviews and content analysis. During the same period, Morris (1999) reported that observational and case studies accounted for just 2% of scientific production in this field between 1979 and 1998.

In a study published shortly afterwards, Biddle et al. (2001) presented a detailed analysis of the methods used in both quantitative and qualitative sport and exercise psychology research, with a focus on discriminant analysis, hierarchical regression, stepwise statistical procedures (although it should be noted that stepwise procedures have been debunked by numerous statisticians; cf. Thompson, 1995; Onwuegbuzie and Daniel, 2003), and meta-analysis in the area of quantitative research and thematic analysis (mostly interviews) in the area of qualitative research. Biddle et al. (2001) words were particularly enlightening:

The extent to which such diverse approaches could or should be integrated is a matter for the reader to decide. Some have stated that qualitative and quantitative approaches reflect fundamentally different paradigms, such as when people refer to qualitative vs. quantitative methods. Although there are obvious differences in the two approaches, there are many cases when the two are combined. (p. 778)

As we will discuss in the last section, one of the main shortcomings of studies that involve an attempt to combine the two approaches is the failure to successfully integrate qualitative and quantitative data. This is consistent with Bazeley's (2010) conclusion that “there are surprisingly few published studies reporting results from projects which make more than very elementary use of the capacity to integrate data and analyses using computers” (p. 434). True integration in applied studies is not easy task, but the aim of this paper is to show how a novel methodological approach grounded within systematic observation can help to overcome some of the challenges involved.

Based on our experience and work, we can now confidently state that the “multifaceted” perspective (Tashakkori and Teddlie, 2010, p. 274) offered by a mixed methods research approach (Johnson et al., 2007) is now widely present in the field of sport and physical activity (van der Roest et al., 2015). An optimal approach would be to take a wide-angle perspective while resisting the temptation to pose an overly broad research question, with the ultimate aim of making future research more effective.

To gain a better perspective on the use of mixed methods research in sports and physical activity studies worldwide, we conducted what Alise and Teddlie (2010) refer to as *prevalence rate* studies, which represents “a line of inquiry into research methods in the social/behavioral sciences [referring to the proportion of articles using a particular methodological approach]” (p. 104), which is undertaken by assessing (a) the prevalence rates of MM [mixed methods] in those fields and (b) the degree to which disciplines are still dominated by the traditional postpositivist QUAN [quantitative] approaches” (p. 107). Specifically, we performed a literature search of ISI-indexed journals in the Web of Science and the ISI Web of Knowledge (Journal Citation Reports) to determine the number of articles applying a mixed methods research approach in this field. We placed no restrictions on language, year, or geographic location.

Table 1 presents a list of the journals analyzed, together with their JCR impact factor and the number of articles that used mixed methods research approaches. They key search term used was *mixed methods* and we did not place any limits on publication dates, although our results show that the majority of articles retrieved were published after the year 2000. Our findings show that, compared with the situation described by Biddle (1997) and Morris (1999), a considerable number of ISI-indexed journals now publish mixed methods research studies. We have included all studies that, based on their keywords, can be considered mixed methods studies from the time the mixed methods movement emerged. The results from the last 15 years highlight the growing number of mixed methods studies published in the field of sport and physical activity. These studies include a considerable number of conceptual and methodological papers on different aspects of mixed methods, which have undoubtedly contributed to the growth of applied empirical studies in this area. Indeed, the 203 mixed methods research articles identified among this set of 67 journals yielded a mean of 3.03 mixed methods research articles (*SD* = 4.98). This represents an important advance, not only because of the increase in studies of this type, but also because it shows that prestigious peer-reviewed journals are now publishing these studies.

**Table 1:** Publication of mixed methods research studies in ISI-Indexed sports and physical activity journals.

**Journal**	**JCR Impact factor**	**Number of Mixed methods articles, no**.
Adapted Physical Activity Quarterly	1.324	2
American Journal of Sports Medicine	4.362	2
British Journal of Sports Medicine	5.025	4
Clinical Journal of Sport Medicine	2.268	2
Current Sports Medicine Reports	1.552	0
European Journal of Sport Science	1.550	2
European Physical Education Review	0.673	12
European Review of Aging and Physical Activity	0.676	0
Exercise and Sport Sciences Reviews	4.252	0
Gait and Posture	2.752	0
Human Movement Science	1.598	0
Health Education Research	1.574	16
International Journal of the History of Sport	0.258	0
International Journal of Performance Analysis in Sport	0.798	1
International Journal of Sport Nutrition and Exercise Metabolism	2.442	0
International Journal of Sport Finance	0.385	0
International Journal of Sport Psychology	0.485	3
International Journal of Sports Medicine	2.065	0
International Journal of Sports Physiology and Performance	2.662	1
International Journal of Sports Science and Coaching	0.480	0
International Review for the Sociology of Sport	0.953	1
International Review of Sport and Exercise Psychology	4.526	0
Isokinetics and Exercise Science	0.488	0
Journal of Aging and Physical Activity	1.966	5
Journal of Applied Biomechanics	0.984	0
Journal of Applied Sport Psychology	1.062	3
Journal of Athletic Training	2.017	7
Journal of Biomechanics	2.751	0
Journal of Electromyography and Kinesiology	1.647	0
Journal of Exercise Science and Fitness	0.333	1
Journal of Human Kinetics	1.029	0
Journal of Motor Behavior	1.418	0
Journal of Physical Activity and Health	2.090	8
Journal of Science and Medicine in Sport	3.194	3
Journal of Sports Science and Medicine	1.025	3
Journal of Sports Sciences	2.246	5
Journal of Teaching in Physical Education	1.021	7
Journal of Science and Medicine in Sport	3.194	3
Journal of Sport and Exercise Psychology	2.185	12
Journal of Sport and Social Issues	0.571	0
Journal of Sport Management	0.718	7
Journal of Sport Rehabilitation	1.276	2
Journal of Sports Medicine and Physical Fitness	0.972	0
Journal of Sports Science and Medicine	1.025	3
Journal of Teaching in Physical Education	1.021	7
Journal of Strength and Conditioning Research	2.075	4
Kinesiology	0.585	1
Medicine and Science in Sports and Exercise	3.983	5
Medicina dello Sport	0.235	0
Motor Control	1.233	0
Pediatric Exercise Science	1.452	0
Perceptual and Motor Skills	0.546	1
Physical Education and Sport Pedagogy	0.811	6
Physical Therapy in Sport	1.653	0
Proceedings of the Institution of Mechanical Engineers Part P-Journal of Sports Engineering and Technology	0.885	0
Psychology of Sport and Exercise	1.896	7
Quality and quantity	0.720	32
Quest	1.017	1
Research in Sports Medicine	1.704	0
Research Quarterly for Exercise and Sport	1.566	9
Revista Internacional de Medicina y Ciencias de la Actividad Fisica y del Deporte	0.146	0
Revista de Psicología del Deporte	0.487	5
Scandinavian Journal of Medicine and Science in Sports	2.896	4
Sociology of Sport Journal	0.750	0
Sport Education and Society	1.288	5
Sports Biomechanics	1.154	0
Sports Medicine	5.038	1
Total Number of Articles	−	203

## Inclusion of purely observational sports and physical activity studies in the field of mixed methods research

Studies in the field of sport and physical activity frequently address immediate research concerns that require a scientific answer to questions related to multiple aspects of learning, training, and performance. Such realities are multifaceted in any field, but we are referring to the specific—and possibly unique—case of studies in which the primary and often the only goal is to capture what is actually happening, with no regard for the administration of standardized tests or the opinions or feelings of the agents involved. Studies in the field of sport and physical activity provide numerous examples of such cases, which, due to their singularity, we believe deserve special consideration (Castañer et al., 2013).

Let us imagine, for example, that we are interested in studying the suitability of a certain tactic in an elite individual or team competition (e.g., a judo or soccer match). A fitting research design would be systematically to observe the athlete's behavior (systematic direct observation) and to conduct an in-depth interview with the athlete and/or his or her trainer after the event (indirect observation). Logically, the responses given by the athlete or trainer might be different to the information portrayed by the video recording (referred by Greene et al., 1989; as *initiation*, which involves discovering paradoxes and contradictions that emerge when findings from the two analytical strands are compared), because opinions regarding performance can understandably vary and can be elaborated on in an interview situation. To meet the goal of our study, we would need to merge the quantitative and qualitative findings by comparing the results of the interview (presuming that these are purely qualitative) with the information captured in the video recordings (as annotation of the behaviors observed in the successive images analyzed produces a systematized, quantifiable dataset built through the coding of data guided by a structured *ad-hoc* observation instrument).

Although interviews as a research method can sometimes raise concerns due, for example, to doubts about sample representativeness (Sandelowski, 1995; Onwuegbuzie, 2003), this is not the case in the example described. The issue of interviews in observational methodology studies of sport and physical activity is very different, and poses more serious questions, as illustrated by the following example.

Let us now imagine that we are studying the fouls committed by an athlete in a competition. If we did not modify our approach, we would be contrasting a visual record of what actually happened with the athlete's interpretation of what happened, with the additional risk that this interpretation could be tainted by considerable cognitive baggage. If the purpose of the study is to analyze the fouls committed by an athlete, what use is it for the athlete to say that he or she did not commit the foul if we have an image showing the contrary? The discrepancies between the two realities could be considerable, both in volume and nature, but that aside, we do not actually need the opinion of the athlete, because the answer to our research question lies in the analysis of fragments of what actually happened. This issue becomes even more complicated if we decide to include quantitative data, such as distances covered, number of steps taken, or heart rate, or if we administer a personality test before and after the competition, because none of this information can shed light on our research question or enrich our findings.

In our opinion, the ideal solution for situations like this (which are very common) is to apply the successive steps defined within observational methodology. These include selecting dimensions and subdimensions designed to answer the research question, taking decisions on segmentation of the observable date into units, proposing a design for each research objective, building a purpose-designed observation instrument, creating a computerized coded dataset that allows the data to be arranged into matrices of codes, checking the reliability and variability of the data collected, and analyzing the behavioral patterns hidden within the code matrices using robust analytical techniques for categorical data. Systematic observation is the main procedure used to collect data in event analysis (Happ et al., 2004) and there is ample experience with its use and evidence of its potential (Anguera, 1979, 2003; Portell et al., 2015b).

The study of spontaneous behavior is characterized by a richness of information that can only be captured by video or sound recordings, without elicitation (Anguera and Hernández-Mendo, 2016), and the possibilities offered in this area have been greatly enhanced by recent technological advances. Examples are (a) integration of data through merging, connecting, and embedding strategies (Plano Clark and Sanders, 2015); (b) integration of multisensor data through data fusion (Liggins et al., 2017), which consists of combining signal- and image-processing techniques with pattern-recognition techniques and artificial intelligence to create multimodal databases; (c) integration of heart rate data captured during exercise with observational data on physical activity through hidden Markov chains (Castañer et al., 2017b); and (d) application of deep learning techniques, which automatically extract multilevel characteristics that maximize the identification of predefined behavioral patterns (Ordóñez and Roggen, 2016). The resulting information is also richer in terms of veracity, as the data are not tainted by a personal opinion but based on an objective recording of what happened.

A careful choice of observation units is a central component of observational research (Anguera and Izquierdo, 2006). The choice of units in the field of sport will be determined by the research question and by the rules of the sport, each with its nuances, and the units must be captured through the careful, rigorous use of video cameras, which is not without its technical complexities. In soccer, for example, a move may be a macro-unit (with the condition that only the team in possession of the ball is observed) but it can also be divided into smaller units depending on, for instance, how a given player establishes contact with the ball or with different team mates or areas of the pitch.

Systematic observation differs from other methods in that the observation instrument must be built *ad-hoc—*that is, it must be purpose-designed in accordance with the theoretical framework of the study. The main instrument used in studies of this type combines a field format system and category systems tailored to the research question (Anguera et al., 2007).

The field format (Sánchez-Algarra and Anguera, 2013) is a multidimensional system. For each field format, it is necessary to draw up a catalog of behaviors (a list of mutually exclusive behaviors for each dimension) that is considered to be permanently open; it is constructed using a decimal coding system that allows the behaviors to be hierarchically arranged according to the degree of molecularization required. The final dataset acquires the form of a matrix of codes consisting of columns containing the different dimensions/subdimensions and rows consisting of the successive units into which the episode observed has been segmented. The category system (Anguera, 2003) is unidimensional and requires a theoretical framework, which, combined with empirical information on the situation being observed, enables the construction of a series of exhaustive, mutually exclusive categories. Instruments that combine field format and category systems aim to harness the strengths of the two systems (flexibility in the first case and support from a theoretical framework in the second) and compensate for their weaknesses (inadequacy of the category system in dynamic processes and multidimensional studies and weakness of the field format system in studies that lack a theoretical framework or in which this framework has been rejected).

Numerous examples have been described in the literature, particularly in recent years, and have been applied to a wide range of sporting contexts, including motor skill analysis (Castañer et al., 2009), physical activity (Castañer et al., 2016b), middle- and long-distance races (Aragón et al., 2015, 2017), basketball (Fernández et al., 2009), soccer (Jonsson et al., 2006; Castañer et al., 2016a, 2017a; Casal et al., 2017; Diana et al., 2017), judo (Gutiérrez-Santiago et al., 2011), hockey (Hernández-Mendo and Anguera, 2002), futsal (Lapresa et al., 2013b), and kinesics (Castañer et al., 2013). *Ad-hoc* instruments have been shown to be equally effective in amateur (Arana et al., 2013) and elite (Barreira et al., 2014) sport. The growing use of combined field-format/category system instruments has undoubtedly has been favored by the increase in observational studies in the field of sport and physical activity. We believe, however, that it is also attributable to the fact that observational methodology is widely applicable and offers an optimal balance between rigor and flexibility.

The number of software programs specifically designed for observational studies has increased in recent years. Apart from general-purpose programs, such as Microsoft Excel and Access, researchers now have access to numerous open-access programs that can be used to record, to display, and to analyze data, as well as to perform quality checks. Our research group has designed several freely accessible software programs to support the scientific community (Hernández-Mendo et al., 2014). Examples are LINCE (Gabin et al., 2012; http://observesport.com), HOISAN (Hernández-Mendo et al., 2012; http://www.menpas.com), MOTS (Castellano et al., 2008; http://www.menpas.com), and SOCCEREYE (Barreira et al., 2013). Another very useful freeware program that our group has been systematically using for years to record observational data and to perform lag sequential analysis is SDIS-GSEG (Bakeman and Quera, 2011).

The concepts and technicalities of quantification (also known as *quantitizing*; Tashakkori and Teddlie, 1998) and data transformation are a recurrent theme in works written by eminent figures in the field of mixed methods research (Sandelowski, 2001; Creswell et al., 2003; Bazeley, 2009b; Sandelowski et al., 2009). Quantification in observational methodology is particularly robust, because apart from simple frequency counts, it contemplates other essential primary parameters, such as order and duration (Bakeman, 1978; Anguera et al., 2001; Bakeman and Quera, 2011), thereby providing the researcher with the means to map the different components of a behavior as it occurs. In observational methodology, the term *progressive order of inclusion* refers to the fact that *frequency* is the parameter that provides the least information; order provides information on *frequency* and *something else* (i.e., sequence of behaviors); and *duration* provides information on *frequency* and *order* (by adding the number of time units for each occurrence of a behavior). This specific consideration of the order parameter is crucial for detecting hidden structures through the quantitative analysis of relationships between different codes in systematized observational datasets.

Precisely because it contains information on order and duration, the initial data set, which is derived from an extremely rich qualitative component, can be analyzed using a wide range of quantitative techniques, producing a set of quantitative results that are then interpreted qualitatively, permitting seamless integration. With observational methodology, we are no longer talking about complementing qualitative and quantitative findings, but rather about integrating them. As stated by Fetters (2016), in his article drawing comparisons between developments in mixed methods research and the transition from the horseless carriage to the modern automobile, innovation is both needed and will occur.

The wide scope of opportunities available for processing data derived from observation supports the idea that purely observational studies should be considered as mixed methods research studies, even though they constitute a somewhat special case and do not follow traditional patterns. Although this is a somewhat controversial topic, as Freshwater (2015, p. 296) stated, “disagreement and debate is fundamental to achieving excellence in scholarship.”

## The way forward: overcoming the shortcomings of integration and symmetry

We are at a critical time for the future of mixed methods research and we believe that the time has come to stop and to take stock, just as we do in our everyday lives, as we come up against different obstacles and challenges. Considering the relatively recent surge in mixed methods research articles around the globe, we believe it is methodologically “healthy” to, as they say in Spain, “put our finger in the blister” and make a humble but firm call for reflection on what we believe to be the two major barriers to the successful implementation of mixed methods research designs: the lack of integration and the lack of symmetry.

### The barrier of integration

Integration of qualitative and quantitative research approaches is a central theme in the mixed methods research literature, and the title of a recent editorial by Fetters and Freshwater (2015b)−1 + 1 = 3—graphically showed that a whole is greater than is the sum of the individual parts. Although it is understandable that researchers from a given discipline typically will follow the traditions of their research communities, it is necessary to bear in mind that the respective findings will be mutually informative—that is, they will *talk to each other* (O'Cathain et al., 2010).

Quantitative methods address questions such as causality, generalizability, and magnitude of effects, whereas qualitative methodologies are used to develop theories, to describe occurrences, and to explore the contexts surrounding different phenomena (Fetters and Freshwater, 2015b). Qualitative data also can be used to design quantitative instruments (Onwuegbuzie et al., 2010). Just like in an orchestra, each of the components in a mixed methods research design has an important role, but the sum of these components form a greater whole. However, as Bazeley (2009b) pointed out in an interesting study that described how different qualitative and quantitative methodologies could be positioned along a continuum, not all types of data or analysis can be integrated.

Although numerous leading figures in the field of mixed methods research have stressed the importance of integrating qualitative and quantitative data (Creswell, 2003, 2015; O'Cathain et al., 2010), a large number of researchers, not surprisingly, still struggle to merge the two approaches and end up publishing their results separately.

We believe that the failure to successfully integrate qualitative and quantitative data is largely due to the nature of the data involved (Bazeley, 2009b) and that this is where we need to focus our efforts, through reflection, inquiry, and exploration of solutions. This lack of data integration might also stem from quantitative and qualitative research questions that are addressed separately within a mixed methods research study (cf. Plano Clark and Badiee, 2010). Qualitative and quantitative data, however, can be integrated using what is known as the *weaving* approach, which involves presenting the respective findings together according to a specific theme or concept. Consequently, we propose that researchers who encounter difficulties merging qualitative and quantitative data in studies of sport and physical activity contemplate an initial exploratory phase in which they search for ways of weaving together their data, at least until a suitable methodological solution is found.

### The barrier of symmetry

Unlike other approaches in experimental studies, which from an enactive framework (Lutz et al., 2002) show the difference between first-person approaches (based on phenomonological data) and third-person approaches (based on physiological and behavioral data obtained objectively using a range of instruments), enabling thus the problem of asymmetry to be overcome, in observational studies of spontaneous behavior in natural settings, where nothing is “artificial” or “staged,” asymmetry acquires a different meaning, as described below.

Mixed methods research studies typically involve the adoption of either a qualitative-dominant or a quantitative-dominant approach (Onwuegbuzie and Combs, 2010), but an additional issue is that studies are frequently characterized by a lack of symmetry between the two approaches. Mixed methods studies typically focus more on qualitative than quantitative data and accordingly miss the opportunity to explore the wealth of information that a quantitative analysis of qualitative data can provide. Although it is true that some researchers apply robust statistical methods and even multiple techniques to analyze quantitative data (Onwuegbuzie et al., 2007), they frequently fail to move beyond a descriptive analysis (Ross and Onwuegbuzie, 2014), and consequently miss out on the opportunity to explore the richness of information within the qualitative component (Bazeley, 2009a; Onwuegbuzie, 2016).

As a step toward achieving this qualitative-quantitative symmetry, we agree with Happ et al. (2004) that it is necessary to quantitize the qualitative data and qualitize the quantitative data using different event analysis techniques, such as, for example, segmenting episodes of behavior into events or measuring duration of behaviors. O'Cathain et al. (2010) also refer to quantitization and qualitization, but argue that it is not sufficient simply to use the qualitative data to inform the quantitative findings, stressing instead the need to mix together the two types of data to create new variables.

Onwuegbuzie et al. (2011) identified 58 types of quantitative analyses, which they grouped into four categories according to level of complexity: number of independent variables, number of dependent variables, measurement scales for independent variables (nominal, ordinal, interval, and ratio scales), and measurements scale for dependent variables. Obviously, each of these categories can be further broken down into additional categories (Ross and Onwuegbuzie, 2014). What we are proposing in this paper, and with specific reference to research in the field of sport and physical activity, is an approach that strengthens the analytical processing of quantitative data derived from the qualitative component of the study. The concept of independent and dependent variables, almost omnipresent in experimental and quasi-experimental studies, is not relevant to systematic observation, because this involves observing spontaneous behaviors in natural settings.

Techniques for analyzing quantitative data obtained from qualitative research are generally complex. Anguera et al. (2014) presented a list of the techniques used in sport and physical activity research. In Table 2, we present an updated version of this list, which also now includes techniques for analyzing data from quantitative sources (Sanchez-Algarra, 2006).

**Table 2:** Quantitative analysis techniques for processing qualitative and quantitative data in studies of sport and physical activity.

**Type of relationship**	**Type of data**	**Quantitative statistical analysis**
Descriptive statistics		Measures of central tendency: Mean Median Mode
		Measures of dispersion: Variance Standard deviation Coefficient of variation
Normal statistical analysis	Quantitative data	*T*-test (one population) *T*-test for comparing means between two groups with independent data *T*-test for comparing means between two groups with paired data *F*-test for equality of variances Univariate analysis of variance (ANOVA): One-way ANOVA Two-way ANOVA (with interaction)
Association	Relationship between two categorical variables	Yule's coefficient (Yule's Q) Contingency coefficient C Chi-square (χ^2^)
	Relationship between several categorical variables	Contingency analysis Log-linear analysis Logit analysis Logit analysis-causal Logit analysis-Markov Probit analysis Logistic regression Correspondence analysis
	Relationship between ordinal variables	Contingency tables Log-linear analysis Logit analysis Correspondence analysis Ballot analysis
	Ratios	Comparison of proportions
	Quantitative data	Pearson's correlation coefficient Simple linear regression Multiple linear regression Partial correlation
Covariance	Relationship between a naturally dichotomous variable and a continuous quantitative variable	Point-biserial correlation coefficient (r_bp_)
	Relationship between an artificially dichotomized variable and a continuous quantitative variable	Point-biserial correlation coefficient (r_b_)
	Relationship between dichotomized variables	Tetrachoric correlation (r_t_)
	Relationship between dichotomous variables	Correlation φ
	Relationship between ordinal variables	Spearman correlation coefficient (r_S_)
		Kendall rank correlation coefficient
		Kendall's W (coefficient of concordance)
	Quantitative data	Product-moment Pearson correlation Simple linear regression model Multiple linear regression model Partial correlation
Multivariate analysis of variance (MANOVA)	Quantitative data	One-way MANOVA Two-way MANOVA (with interaction)
Statistical distances and dimension reduction	Quantitative data	Principal component analysis Calculation of number of principal components Geometric interpretation
	Qualitative data	Principal coordinate analysis (multidimensional scaling) Algorithm for calculating principal coordinates
Discriminant analysis	Quantitative data	Main classification algorithms: Discriminant analysis. Fisher linear discriminant analysis and quadratic discriminant analysis: Minimum or maximum method; Unweighted Pair-Groups Method Average (UPGMA) method. Cophenetic correlation.
Canonical correlation analysis	Quantitative data	Population canonical correlation analysis Euclidean distance and Mahalanobis distance
Sequentiality	Ordered categorical data analysis	Lag sequential analysis
		Polar coordinate analysis
		T-pattern detection (temporal patterns)
	Quantitative data	Time series analysis Spectral analysis

Table 2 shows the wide range of possibilities that exist for analyzing the qualitative and quantitative data that coexist in observational studies in the field of sport and physical activity. As the table demonstrates, however, what is novel about our approach from a mixed methods perspective is the way in which we integrate or mix the two types of data. Generally speaking, “there are three ways in which mixing occurs: merging or converging the two datasets by actually bringing them together, connecting the two datasets by having one build on the other, or embedding one data set within the other so that one type of data provides a supportive role for the other data set” (Creswell and Plano Clark, 2007, p. 7). For our proposal, we chose the second form: connecting two databases by having one build on the other. According to Sandelowski et al. (2009), this connection can be achieved through transformation, i.e., by quantitizing qualitative data or by qualitizing quantitative data. We use the sequentiality method, shown in the last row in Table 2, which takes as its starting point the annotation of the order of occurrence of all the behaviors included in a given observational dataset. This sequentiality permits the transformation of initially qualitative data into a format that can be analyzed quantitatively and robustly, achieving thus successful integration.

None of the standard research designs conceptualized for mixed methods research are applicable to the transformation of qualitative data (derived from video or sound recordings in natural settings, or from texts resulting from indirect observation) into quantitative data for analysis using specific quantitative techniques, such as variability analysis, comparison of proportions, categorical variance, log-linear analysis, logit analysis, lag sequential analysis, polar coordinate analysis, T-pattern detection, and so forth. Perhaps the use of such techniques will enable the weaving approach called for in mixed methods research. Several data analysis techniques that are specific to the study of sequences of behavior, such as lag sequential analysis, polar coordinate analysis, and T-pattern detection, have a particularly important role in observational methodology due to the assignment of parameters of frequency, order, and duration to the initial qualitative data (Anguera et al., 2001; Blanco-Villaseñor et al., 2003) and thereby providing the necessary conditions for subsequent quantitative analysis using robust, non-standard, statistical techniques that offer highly relevant structural results.

As an epilog, we would like to stress that there is wide consensus in the mixed methods research field on the value of using merging, connecting, and embedding strategies to integrate qualitative and quantitative data (Plano Clark and Sanders, 2015). In this article, we have focused on an approach for connecting these two perspectives and shown that it is perfectly possible to transform qualitative datasets featuring behaviors whose order of occurrence has been recorded into matrices of code that can subsequently be analyzed using powerful quantitative techniques.

## Discussion

The scientific literature of the past 25 years has presented, from varying and sometimes opposing standpoints, a wide spectrum of theoretical positions and empirical findings in relation to studies that are claimed to represent mixed methods research studies (López-Fernández and Molina-Azorín, 2011). Mixed methods research is growing in most disciplines—as demonstrated by Onwuegbuzie and Corrigan (2016) via their recent meta-prevalence rate study. And, in our opinion, the field of sport and physical activity is a particularly fertile area in which studies merging quantitative and qualitative approaches have begun to flourish. These studies typically involve the analysis of behaviors and motor-related skills in a wide range of sports and activities that are grounded in a theoretical framework, can be performed at a professional or amateur level, offer big learning/training opportunities due to their vast scope, and have the potential for causing considerable impact in the scientific community and media at large. They are, as such, particularly deserving of attention. As shown by our review of the literature, summarized in Table 1, the volume of mixed methods research publications in ISI-indexed sports and physical activity journals varies widely from one journal to the next. Perhaps, as Fetters (2016) suggests via his analogy of the horseless carriage, we were unaware that the many decades of *tinkering* with mixed methods would spawn a period of accelerated development. We should also, however, bear in mind the saying “do not put vintage wine into new wineskins lest it sour.” That stated, although these “wineskins” are new, they will have benefited from the experience gradually accumulated in multiple substantive areas over the past two decades.

As the guiding principle of this paper was to show the specificities of observational studies in the field of sport and physical activity, it is only logical that we also critically appraise alternative options described in the scientific literature. With reference to studies based on first-person and third-person descriptions, it is important to note that the former refer to “lived experiences” linked to cognitive and mental events, while the latter refer to descriptive experiences linked to the study of other natural phenomena. Both, however, can be connected using a phenomenological approach. In an experimental setting designed to analyze processes, such as attention or memory, for example, subjects are asked to perform a specific task but while doing this, they experience what can be termed “lived content.” As this is something that can be described and analyzed, it exists. What sets our proposal apart is that we always analyze spontaneous behavior in natural settings. The subject is given no instructions, as the setting is natural, not artificial. Consequently, despite the vitality of lived experiences and the issues regarding the what, why, and how of first-person methodologies (Varela and Shear, 1999a,b), the start and end points of first- and third-person methodologies are different, although it is perfectly possible to integrate qualitative and quantitative data in both approaches if there is sufficient symmetry.

One interesting point for reflection was recently proposed by Depraz et al. (2017), who addressed the challenges associated with generating productive interaction between first-person data (in this case micro-phenomenological interviews) and third-person data (objective physiological data) by co-interpreting both analyses (each from the other) using an approach based on “co-validation” and “mutual constraints” (Varela, 1996). Their proposal was discussed in depth in subsequent open peer commentaries (also available in Varela, 1996). In our field, reflection on the issues they discuss could give rise to a line of research focused on the perceivable expression of spontaneous behaviors, which would influence the categorization stage of our method.

We hope that the continued development of research within systematic observation will trigger reflection and inquiry and contribute to the creation of knowledge in the field of sport and physical activity. Systematic observation has commendable strengths, but it also requires a certain *sacrifice* in terms of time, labor, and pursuit of scientific rigor. The strength of systematic observation is that it provides a flexible yet rigorous framework for the objective analysis of behaviors in a natural setting and context; this is the only way to study spontaneous behaviors in a natural environment. In this article, we have discussed the essential role of observational studies in this respect and highlighted the benefits of transforming rigorously annotated, sequential (ordered) qualitative data into code matrices for subsequent analysis using powerful quantitative analytical techniques. Systematic observation in its current form permits the seamless integration of qualitative and quantitative data (Sánchez-Algarra and Anguera, 2013), while meeting the requirements of multi-method research studies and harnessing the potential offered by the quantitization of qualitative data using non-standard statistical techniques and the subsequent *interpretive* qualitization of the quantitative results (which are largely structural) using behavior sequence analysis techniques.

Finally, we would like to express our concern that the fundamental meaning of mixed methods research, at times, has been misunderstood, leading to the publication of *pseudo-studies* in the form of qualitative research supplemented by some quantitative results or quantitative research adorned with a qualitative component, such as an interview or autobiographical text. We believe that the unorthodox use and understanding of the term *mixed methods* has generated confusion that has not been adequately addressed. The literature contains many examples of studies that the authors claimed to represent mixed methods that are not, but it also contains studies that are mixed methods research studies but whose findings are limited by a lack of integration and/or symmetry. The difficulty of integrating qualitative and quantitative research approaches has been widely acknowledged (Fetters and Freshwater, 2015b) and is largely linked to a lack of a strong scientific culture. Achieving the symmetry between the two approaches will be a difficult process because they originate from very different traditions. We are aware of the difficulties that lie ahead in our field but we believe that we need to move forward and to assume the responsibility of providing methodological training focused precisely on these two weak points: integration and symmetry.

To conclude, we would like to acknowledge the initiative shown by Fetters and Freshwater (2015a) in calling for contributions that stimulate open reflection and dialog among researchers from multiple disciplines interested in helping to define and to conceptualize mixed methods research. We, for one, are very interested in engaging in this dialog.

## Author contributions

All authors contributed to documenting, drafting and writing the manuscript, and gave their approval to the final version to be published.

### Conflict of interest statement

The authors declare that the research was conducted in the absence of any commercial or financial relationships that could be construed as a potential conflict of interest.
